# A Systematic Review of the Accuracy of Crowns Designed Using Artificial Intelligence Versus CAD/CAM and Traditional Methods

**DOI:** 10.3390/medicina62030567

**Published:** 2026-03-18

**Authors:** Mohammed A. Alfaifi

**Affiliations:** Department of Prosthetic Dental Sciences, King Khalid University College of Dentistry, Abha 62529, Saudi Arabia; mualfaifi@kku.edu.sa

**Keywords:** artificial intelligence, CAD-CAM, marginal fit, accuracy, occlusal contact, internal fit

## Abstract

*Background and Objectives:* Advances in digital dentistry, particularly CAD-CAM, have improved the efficiency and precision of crown design and fabrication. Recently, artificial intelligence (AI)-integrated CAD-CAM systems have enabled automated tooth morphology generation, margin detection, and occlusal analysis, enhancing consistency and accuracy. This systematic review evaluates the accuracy of AI-assisted crown design compared with conventional and CAD-CAM workflows. *Materials and Methods:* A systematic search was conducted across PubMed/MEDLINE, Scopus, Web of Science, Cochrane, and LILACS for studies published between January 2010 and December 2025 that assessed the marginal fit, internal adaptation, and occlusal contact accuracy of single crowns. Screening, full-text assessment, and data extraction followed Preferred Reporting Items for Systematic Reviews and Meta-Analyses (PRISMA) guidelines. Methodological quality and risk of bias were evaluated using the Modified CONSORT checklist for in vitro studies and the Joanna Briggs Institute tools for clinical studies. *Results:* Of 887 records identified, 12 studies met the inclusion criteria. Nine studies showed a moderate risk of bias, two moderate-to-high, and one low-to-moderate. AI-assisted crown design demonstrated clinically acceptable internal fit and marginal adaptation, comparable or superior to CAD-CAM systems. Occlusal contact accuracy was generally comparable to CAD-CAM and technician-designed crowns, though variability was observed across AI models. *Conclusions:* AI-assisted crown design provides a reliable fit and marginal adaptation, with occlusal accuracy approaching conventional CAD-CAM and technician workflows. While not a replacement for clinical expertise, AI serves as a valuable adjunct, enhancing reproducibility, precision, and overall quality in restorative dentistry. Further standardized clinical studies are needed to validate long-term outcomes and optimize occlusal performance.

## 1. Introduction

Over the past several years, transformative advances in dental science, driven by groundbreaking innovations, have reshaped contemporary dental practices. Advancements in the fundamental sciences underlying oral health are driving the expansion of personalized care, supported by robust scientific evidence, particularly in prosthodontics and implantology [1,2].

Single-crown restorations are commonly used in prosthetic dentistry to restore lost tooth structure, recover function, and achieve esthetic rehabilitation. For predictable clinical success, these restorations must exhibit optimal integration with biological, mechanical, and esthetic requirements [3]. While conventional techniques for manufacturing these crowns have provided acceptable clinical outcomes for many years, the introduction of digital dentistry has enabled workflows that enhance accuracy, efficiency, and procedural simplicity while reducing clinical time. As a result, digital methods are increasingly considered to offer greater potential than conventional techniques [4,5,6]. One such innovation in the realm of prosthetic dentistry is the incorporation of CAD-CAM for crown designing and manufacturing. A major advantage of CAD/CAM workflows in prosthodontics is the reduction in clinical chairside time, together with the minimization of inaccuracies associated with conventional impression techniques and laboratory processing. These digital workflows facilitate the fabrication of restorations with improved marginal and internal fit, enhanced precision, and favorable long-term clinical performance, thereby increasing patient satisfaction. Furthermore, the digital storage of patient data enables the efficient reproduction or remanufacture of prosthetic restorations when required [7,8].

CAD/CAM design of dental crowns involves a digital impression of the prepared tooth and surrounding teeth via an intraoral scanner or direct scanning of the dental cast containing the prepared tooth, which creates a precise 3D representation. These data are then transferred to CAD (computer-aided design) software, where a virtual crown is designed. The software allows the customization of occlusion, contacts, and morphology, and may use design modes such as bio generic copy, bio generic individual, or bio generic reference depending on the system. Once the design is finalized, it is sent to a CAM (Computer-Aided Manufacturing) machine for fabrication, either by milling the crown from ceramic or zirconia blocks or by 3D printing. For certain materials, such as zirconia, the crown undergoes sintering to achieve final strength, followed by polishing, glazing, or staining to improve esthetics [9,10].

Multiple studies evaluating crown fabrication have shown that digital workflows are generally faster and more convenient for patients [11,12]. Additionally, many clinical studies have compared the accuracy and fit of crowns designed conventionally and via CAD-CAM. In a study by Alqahtani F [13] on the marginal fit of all-ceramic crowns fabricated using CAD/CAM systems compared with the conventional technique, it was concluded that CAD/CAM-fabricated crowns had the smallest marginal gap. According to in vivo clinical research comparing digital scanning and CAD/CAM castings with conventional impressions for implant-supported restorations, digital procedures were more efficient and had equivalent accuracy to conventional approaches, with most participants preferring digital scans [14]. In another study by Abdullah et al., in which CAD/CAM provisional crowns were compared with direct conventional provisional crowns, it was concluded that CAD/CAM provisional crowns demonstrated superior marginal fit, with the mean internal gap lower than that of direct provisional crowns [15].

Although traditional CAD/CAM systems have revolutionized dental crown production, delivering exact and predictable results, they still rely on manual input and technician knowledge for intricate crown design, which can increase design time and introduce variability across operators. Also, conventional CAD/CAM lacks advanced pattern recognition and data-driven optimization, which can make it slower and less adaptable to complex anatomical variations [16]. To overcome this, artificial intelligence (AI)-based designing of crowns has recently evolved.

AI refers to the capability of computers to perform tasks that normally require human intelligence, such as learning, reasoning, and problem-solving [17]. AI applications rely on machine learning, a method in which mathematical models are trained to identify statistical patterns within data to make predictions. A specialized branch of machine learning, deep learning, uses multi-layered neural networks with complex architectures, enabling these algorithms to outperform conventional machine learning approaches at detecting patterns in large, diverse datasets. The use of AI demonstrates significant potential across multiple diagnostic [18] and treatment approaches in prosthetic dentistry, especially crown designing [19]. It can enhance efficiency and standardization beyond what traditional CAD/CAM achieves, potentially minimizing design inconsistencies and reducing human error [20].

Both in vitro and in vivo studies with diverse findings have compared AI-based dental crown design with conventional techniques and traditional CAD-CAM workflows. Meshram et al. reported in a comparative clinical study that AI-designed crowns exhibited improved internal and marginal adaptation compared with crowns produced with conventional CAD software [21]. In vitro studies by Wu et al. [22] and Chen et al. [23] found that although AI-powered software improves crown design efficiency, it shows similar or inferior morphological performance to technician-driven CAD workflows, indicating the need for more advanced data-driven modeling.

Based on the available literature, a systematic review directly comparing the efficiency of CAD/CAM, conventional, and AI-based design workflows in terms of marginal fit and accuracy appears to be lacking. Accordingly, the present systematic review is conducted to comprehensively compare and evaluate these findings. Thus, the null hypothesis was formulated that there is no significant difference in the marginal fit, internal fit, and occlusal contact accuracy of single crowns fabricated using AI-based design approaches compared with CAD/CAM and conventional techniques.

## 2. Materials and Methods

### 2.1. Registration Protocol

The current systematic review was submitted to the International Prospective Register of Systematic Reviews (PROSPERO) under registration number CRD420261281643 and was conducted in accordance with PRISMA criteria [24,25] (Table S1).

### 2.2. Research Question and Eligibility Criteria

The main review question was, “Do crowns designed using AI-based systems demonstrate superior or comparable accuracy and clinical performance, in terms of marginal and internal adaptation, and occlusal contact accuracy, when compared with crowns designed using conventional CAD–CAM software and dental technician-based workflows?” The focus question was formulated using the Population, Intervention, Comparator, Outcome, and Study (PICOS) framework, as summarized in Table 1, followed by a predefined search strategy that outlined the inclusion and exclusion criteria, study identification and selection, quality assessment, data extraction, evidence table construction, and result interpretation.

The inclusion criteria involved the inclusion of articles/studies published in the English language from 1 January 2010 to 31 December 2025, evaluating and/or comparing the marginal and/or internal fit/adaptation and occlusal contact accuracy of single crowns designed via AI, CAD-CAM, and technician (conventional procedure). Case reports, case series, review articles, animal studies, non-English publications, studies published before 1 January 2010, investigations assessing outcomes outside the predefined inclusion criteria, and studies evaluating prostheses other than single crowns were excluded from the review.

### 2.3. Search Strategy, Study Selection, and Data Extraction

The primary electronic databases searched included CENTRAL (Cochrane Central Register of Controlled Trials), LILACS (Latin American and Caribbean Health Sciences Literature), MEDLINE/PubMed, Scopus, and Web of Science, supplemented by manual reference screening and citation analysis. For data search, the appropriate Boolean operators (AND, OR, and NOT) were utilized to combine keywords and medical subject heading terms (MeSH) phrases as listed in Table 2. Two researchers (M.A and H.A.) independently removed duplicate articles and reviewed titles and abstracts to remove irrelevant titles. Following the initial search, they assessed the full-text articles of all eligible studies. Any disagreements were resolved by discussion with a third researcher (Ma. A.). Later, they extracted the relevant data, which are presented in Table 3 and Table 4.

### 2.4. Synthesis of Results

A qualitative (narrative) synthesis was performed to summarize the findings of the included studies. Studies were grouped by comparison type (AI-based design vs conventional CAD-CAM design or technician-based design) and outcome domain (marginal fit, internal fit, and occlusal contact accuracy). Extracted outcome data were tabulated and compared descriptively across studies. Meta-analysis was not conducted due to heterogeneity in study designs (in vitro [16,26,27,29,30,31,32,33,34,35] and in vivo [37,38]), differences in crown materials (Cercon system, IPS e.max Press, and metal–ceramic restorations, PMMA, lithium disilicate), and fabrication techniques (variability in measurement methods (e.g., triple-scan, replica technique)), and inconsistent reporting of outcome parameters. Therefore, results were synthesized narratively to provide a structured comparison of outcomes across studies.

### 2.5. Quality Assessment

Two independent reviewers (M. A. and Ma. A.) assessed the methodological quality and risk of bias using the Modified CONSORT checklist for in vitro studies and the Joanna Briggs Institute (JBI) critical appraisal tools for in vivo studies [37,38]. Studies were classified as having a low risk of bias when more than 70% of appraisal items were scored as “yes,” a moderate risk of bias when “yes” scores ranged from 50% to 69%, and a high risk of bias when “yes” scores were ≤49% for each article [38,39]. Inter-reviewer agreement was evaluated using Cohen’s kappa, yielding a coefficient of 0.86 for full-text screening, indicating strong agreement between the reviewers [40].

Risk of bias due to missing results (publication bias) was assessed qualitatively by evaluating selective outcome reporting and inconsistencies between study objectives and reported results. Funnel plot analysis was not performed because the number of included studies per outcome was insufficient for meaningful statistical assessment. Additionally, publication bias was considered during the GRADE certainty assessment.

### 2.6. Assessment of Strength of Evidence

The certainty of the body of evidence for each key outcome (marginal fit, internal fit, and occlusal contact points) was evaluated using the Grading of Recommendations Assessment, Development and Evaluation (GRADE) approach. The quality of evidence was rated as high, moderate, low, or very low based on five domains: risk of bias, inconsistency, indirectness, imprecision, and publication bias. Evidence from randomized clinical trials was initially considered high-quality, whereas evidence from non-randomized clinical studies and in vitro studies was initially considered low-quality. The final certainty ratings were summarized in a GRADE evidence profile table [41].

## 3. Results

### 3.1. Identification and Screening

**Figure 1:**
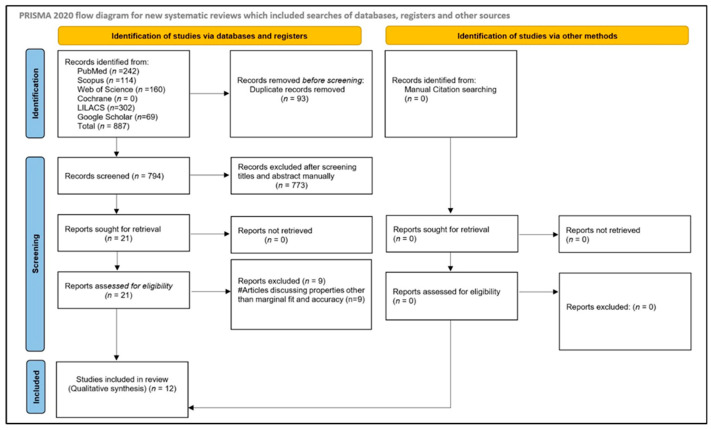
Article selection strategy based on PRISMA guidelines.

The initial electronic and manual searches identified 887 records. After removing duplicates, 794 articles remained for screening. Title and abstract screening yielded 21 potentially relevant studies. Full-text assessment of these articles was subsequently performed, and reference lists were hand-searched to identify additional eligible studies; however, no further relevant articles were found. Among the assessed full-text articles, nine were excluded for various reasons. Following risk-of-bias assessment, 12 studies [16,26,27,28,29,30,31,32,33,34,35,36] were ultimately included in the qualitative synthesis: 10 in vitro studies [16,26,27,29,30,31,32,33,34,35], one prospective study [36], and one randomized controlled trial (RCT) [26]. The study selection process is illustrated in Figure 1.

A quantitative meta-analysis was not performed due to substantial heterogeneity among the included studies in study design (in vitro vs. clinical), evaluation methods (micro-CT, silicone replica, 3D superimposition, triple-scan), outcome measurement units, and reporting formats. Therefore, findings were synthesized qualitatively.

### 3.2. Quality Assessment of Included Studies

Risk-of-bias assessment was performed using the Modified CONSORT checklist for the 10 in vitro studies [21,27,29,30,31,32,33,34,35], which is depicted in Table 5, while the JBI critical appraisal tools were applied to the remaining two studies [28,36]. Among the in vitro studies, eight [29,30,31,32,33,34,35] demonstrated a moderate risk of bias, whereas two [26,27] exhibited a moderate-to-high risk of bias. Of the two studies assessed using the JBI tools, one [28] showed a moderate risk of bias, and the other [36] demonstrated a low risk of bias, as depicted in Table 6 and Table 7, respectively.

### 3.3. Quality of Evidence (GRADE Evaluation)

The certainty of evidence was rated as moderate for marginal, internal fit and occlusal contact outcomes using the GRADE approach, which is summarized in Table 8. This was mainly due to a risk of bias, indirectness resulting from predominantly in vitro evidence, and imprecision related to small sample sizes and measurement variability. Additionally, inconsistency in occlusal findings and suspected publication bias further reduced confidence in the overall effect estimates.

**Table 8:** Assessment of strength of evidence (GRADE evaluation).

S.No.	Outcomes Evaluated	Inconsistency	Indirectness	Imprecision	Risk of Bias	Publication Bias	Strength of Evidence
**1**	**Marginal Fit**	Not Present	Not Present	Not Present	Present	Suspected	Moderate (⬤⬤⬤◯)
**2**	**Internal Fit**	Not Present	Not Present	Not Present	Present	Suspected	Moderate (⬤⬤⬤◯)
**3**	**Occlusal Contact Points**	Not Present	Not Present	Not Present	Present	Suspected	Moderate (⬤⬤⬤◯)

### 3.4. Overview of Included Studies

Of the 12 studies [16,26,27,28,29,30,31,32,33,34,35,36] included in the qualitative synthesis, three [26,33,35] evaluated and/or compared the marginal and/or internal fit/adaptation and occlusal contact accuracy of implant-supported single crowns fabricated using AI-based systems, CAD/CAM workflows, and conventional technician-driven procedures. Three studies [28,34,36] assessed these outcomes in provisional tooth-supported crowns, while six studies [27,29,30,31,32] evaluated permanent tooth-supported crowns. Among the 12 included studies, seven studies [16,29,30,32,33,34,36] compared the evaluated properties of crowns designed using AI-based systems and CAD/CAM workflows, while one study [35] compared crowns designed using AI-based systems, CAD/CAM workflows, and conventional technician-driven procedures, in accordance with the inclusion criteria.

Overall, 1630 crowns were designed using artificial intelligence-based systems, CAD/CAM workflows, or conventional procedures. Of these, 60 were implant-supported single crowns, 194 were provisional tooth-supported crowns, and 1376 were permanent tooth-supported crowns.

For the assessment of marginal and/or internal fit, Nejatidanesh et al. [26] and Bae et al. [31] employed the silicone replica technique, while Win et al. [36] used the triple-scan technique. Mostafa et al. [27] utilized micro-CT, whereas the remaining studies used 3D superimposition software to analyze marginal and/or internal fit and occlusal contact accuracy [16,28,29,30,32,33,34,35].

### 3.5. Marginal Fit/Accuracy and Internal Fit

When comparing the internal fit of single crowns designed using AI-based systems, CAD/CAM workflows, and conventional procedures, Cho et al. [29] and Kızılkaya et al. [34] reported that AI-designed crowns exhibited superior marginal and internal fit compared with those fabricated using CAD/CAM workflows. In contrast, Nagata et al. [16], Cho et al. [32], and Win et al. [36] reported comparable internal fit between AI- and CAD/CAM-designed crowns. Regarding marginal fit/accuracy, both Nagata et al. [16] and Win et al. [36] found no statistically significant differences between crowns designed with AI-based systems and those produced via CAD/CAM workflows. Furthermore, when CAD/CAM-designed crowns were compared with those produced using conventional techniques, CAD/CAM crowns demonstrated superior marginal and internal fit.

### 3.6. Occlusal Contact Accuracy

When comparing the occlusal contact accuracy of single crowns designed using AI-based systems, CAD/CAM workflows, and conventional procedures, Ding et al. [30] and Cho et al. [32] reported comparable occlusal contact accuracy between AI- and CAD/CAM-designed crowns. In contrast, Nagata et al. [16], Cho et al. [33], and Win et al. [36] reported superior occlusal contact accuracy with AI-designed crowns. Ren et al. [35] concluded that the occlusal contact accuracy of AI-designed crowns was comparable to that of technician-designed crowns and superior to CAD/CAM-designed crowns. Additionally, Cheng et al. [28] reported that CAD/CAM-designed crowns demonstrated superior occlusal contact accuracy compared with technician-designed crowns.

## 4. Discussion

Based on the synthesized data from the included studies, the null hypothesis—there is no significant difference in the marginal fit, internal fit, and occlusal contact accuracy of single crowns fabricated using conventional techniques, CAD/CAM systems, or AI-based design approaches—was partially rejected. The evaluated outcomes demonstrated variability across studies, with AI-based methods yielding results comparable to, and in some cases superior to, those obtained with CAD/CAM and conventional fabrication techniques.

In the present review, AI-based dental crown design was assessed and compared with CAD/CAM and conventional fabrication techniques to validate its accuracy, reliability, and clinical applicability relative to established workflows. This comparative evaluation was conducted to assess the added value and reproducibility of AI-based systems, evaluate their potential to enhance restorative outcomes, and provide evidence to support their integration into contemporary prosthodontic practice [42].

The fit and accuracy of dental crowns can be assessed using the silicone replica technique, micro-CT, cross-sectional method (CSM), triple-scan method (TSM), optical coherence tomography (OCT), and 3-dimensional (3D) superimposition software [43]. Therefore, studies utilizing these methods of fit assessment were included in the review. The method used to assess marginal and internal fit also influenced the reported outcomes. Micro-CT, in particular, is considered the most accurate method due to its non-destructive, high-resolution, 3D assessment of the entire crown–tooth interface [44]. Studies employing 3D techniques such as micro-CT and digital superimposition provide more comprehensive and accurate evaluation than two-dimensional (2D) methods such as the silicone replica technique [45,46].

In this review, the accuracy of AI-designed crowns was evaluated for marginal fit, internal fit, and occlusal contact accuracy, and these outcomes were compared with those obtained with CAD/CAM systems and technician-based workflows. To assess the efficiency of a crown/prosthesis manufacturing procedure, its fit and accuracy are key parameters that contribute to the long-term success of restorative treatments [45,46]. Marginal fit refers to the degree of adaptation between the restoration and the finish line of the tooth preparation. In contrast, internal fit describes the quality of contact between the internal surfaces of the restoration and the prepared tooth structure [44,47]. Inadequate marginal and internal fit can result in cement dissolution, marginal staining, microleakage, and the development of secondary caries, gingival inflammation, pulpal inflammation/necrosis, decreased fracture strength, and decreased bond strength, along with premature dislodgement of the restoration. Consequently, minimizing marginal discrepancies is essential to reduce the risk of these complications and enhance the longevity of restorations [48,49,50].

Consequently, studies assessing internal and marginal fit were included in this review.

Across the included studies, AI-designed crowns consistently demonstrated marginal and internal fit values within clinically acceptable limits (≤120 µm), comparable to CAD/CAM-fabricated restorations [16,29,32]. According to the findings of the present review, Nagata et al. [16], Cho et al. [32], and Win et al. [36] reported that AI-designed crowns exhibited internal fit comparable to that of crowns fabricated using CAD/CAM workflows. Also, a few included studies concluded that AI-based crowns have a better internal fit as compared to CAD-CAM-designed crowns, especially on the buccal and distal surface [29,34], which is in accordance with the study conducted by Meshram et al. [21]. The probable reason for this would be that the buccal and distal surfaces exhibit greater anatomical complexity, which may limit the performance of rule-based CAD-CAM offset algorithms. In contrast, AI-based design systems trained on large datasets can better model complex surface anatomy, potentially reducing internal gaps in these regions [29,50]. The variability among study outcomes may be attributed to differences in AI architectures, training datasets, cement space parameters, and assessment methodologies [16,32,36].

Regarding marginal fit, most studies found no statistically significant differences between AI-based and CAD/CAM-designed crowns [16,36]. This finding suggests that AI-assisted design does not compromise marginal integrity and performs at least as reliably as conventional digital workflows. This may be attributed to the fact that AI workflow enables automated identification of the prepared abutment tooth via a preparation tooth extractor module, followed by intaglio surface generation, parametric surface modeling, undercut elimination, and application of multivariable internal offsets through an inner surface generator module. This advanced, data-driven design strategy represents a promising alternative to conventional CAD approaches, providing a reliable and efficient method for dental prosthesis design [29,51].

Apart from marginal and internal fit, occlusal contact accuracy is also a key functional parameter in prosthodontics, reflecting how closely a fabricated crown replicates the natural occlusal scheme and distributes occlusal load. Ideal occlusal contacts minimize the need for chairside adjustments, contribute to even force distribution during mastication, and reduce the risk of premature contact, wear, or temporomandibular dysfunction [52]. Across the included studies, AI-designed crowns showed variable yet generally favorable occlusal contact performance when compared with CAD/CAM and technician-designed crowns. Some studies included in this review, such as Ding et al. [30] and Cho et al. [32], reported comparable occlusal contact accuracy between AI-assisted and CAD/CAM approaches, suggesting that AI systems can generate occlusal morphology with functional accuracy equivalent to that of traditional digital workflows. This aligns with the study by Liu et al. [53], which concluded that AI-designed crowns demonstrated occlusal contact characteristics comparable to those of CAD-CAM-designed crowns. Although spatial differences in contact distribution were observed between the two approaches, both in vivo and in vitro evaluations indicated clinically favorable realism and contact quality. Conversely, other studies reported superior occlusal outcomes with AI-designed crowns. Nagata et al. [16], Cho et al. [33], and Win et al. [36] observed that AI-generated crowns exhibited more precise occlusal contact distributions with fewer premature contacts than CAD/CAM designs, implying improved functional adaptation and potentially reduced clinical adjustment time. This improved occlusal contact precision of AI-generated crowns may be attributed to data-driven occlusal modeling that dynamically optimizes cusp morphology and contact distribution, in contrast to the rule-based algorithms used in conventional CAD/CAM systems [16,29]. Ren et al. [35] further demonstrated that AI-designed implant-supported crowns produced occlusal patterns closely resembling those of technician-designed restorations, with fewer premature contacts than standard CAD/CAM crowns. This aligns with a study by Gu et al. [54], which concluded that AI-generated crowns showed a mean deviation of 0.180 mm from expert designs, outperforming CAD methods in occlusal detail and functional surface accuracy. In another study, Wang et al. [55] concluded that occlusal contact analysis showed no statistically significant differences between AI-designed and technician-designed crowns, despite variability in the number and area of contact points. The similarity between AI-designed and technician-designed occlusal patterns may be attributed to AI systems’ data-driven learning capabilities, which enable the reproduction of complex occlusal morphology and contact distribution derived from expert-designed datasets. Unlike template-based CAD approaches, AI models generate individualized occlusal surfaces that more closely reflect functional anatomy, thereby approximating technician-level design outcomes [42].

With the rapid expansion of artificial intelligence across all areas of dentistry, it is important not to overlook the limitations and challenges associated with AI integration. Research has shown that AI should be implemented cautiously in clinical dentistry, with strict monitoring and regulatory protocols needed to address ethical concerns [56,57]. Studies have identified a lack of transparency in algorithms, risks of bias in data training, and challenges in managing personal health data [56,57,58]. Clear parameters must be set for data security, algorithm transparency, and the delineation of clinical accountability.

Overall, the evidence suggests that AI-based crown design is a reliable and clinically acceptable alternative to CAD/CAM and conventional workflows. AI systems offer the potential to standardize crown morphology, reduce operator dependency, and optimize internal and occlusal fit. These technological advantages may enhance reproducibility, reduce clinical adjustment requirements, and support the broader integration of AI-assisted workflows in restorative dentistry. Nevertheless, the predominance of in vitro studies and the heterogeneity of study designs highlight the need for well-designed, long-term clinical trials to validate these findings and determine their impact on patient-centered outcomes, such as restoration longevity, function, and satisfaction.

## 5. Conclusions

The findings of this systematic review indicate that AI-based crown design systems achieve clinically acceptable accuracy, with internal fit and marginal adaptation comparable to, and in several studies superior to, those of conventional CAD-CAM design workflows. Across the included studies, AI- and DL-assisted approaches generally produced occlusal contact characteristics comparable to those of technician-designed and conventional CAD-CAM crowns, often resulting in reduced need for chairside occlusal adjustment. Nevertheless, variability in occlusal accuracy was observed across AI models, with some generative AI systems demonstrating greater occlusal discrepancies despite acceptable overall fit. Clinical relevance includes a shorter fabrication time and greater predictability and accuracy, especially for less experienced dentists. Collectively, the evidence supports AI’s potential as a reliable and effective tool for crown design, while underscoring the need for well-designed clinical studies with standardized outcome measures to confirm long-term clinical performance and optimize occlusal accuracy.

## Figures and Tables

**Table 1:** PICOS strategy used in the study.

Elements	Contents
Population (P)	• Studies evaluating dental crowns designed using artificial intelligence (AI)-based systems, including machine learning or deep learning approaches; • Studies involving in vitro models (digital or physical models) or clinical settings where AI-designed crowns are assessed; • Studies reporting accuracy-related outcomes, such as marginal fit, internal fit, trueness, precision and occlusal contact accuracy.
Intervention (I)	AI-based dental crowns.
Comparator (C)	CAD-CAM-designed crowns and technician-designed crowns.
Outcome (O)	Marginal and internal adaptation/fit, occlusal contact accuracy.
Studies (S)	Randomized controlled trial (RCT), experimental studies, observation studies.

**Table 2:** Search strings utilized across the assessed databases.

Database	Search Strings
**PubMed**	(Artificial intelligence) AND (dental crowns); (((deep learning) AND (dental crown)) AND (design)) AND (accuracy); (((deep learning) AND (dental crown)) AND (design)); ((Deep Learning) AND (Dental Crown designing)) AND (Accuracy); ((Marginal fit) AND (Artificial Intelligence)) AND (Dental Crowns); (((((artificial intelligence) OR (machine learning)) AND (Dental Crowns)) NOT (implant crowns)) NOT (endo crowns)) NOT (systematic review); (((((neural network) OR (automated design)) AND (dental crowns)) NOT (implant crowns)) NOT (systematic reviews)) NOT (endocrowns) ((CAD-CAM) AND (Marginal accuracy)) AND (Dental Crown); (((CAD CAM) AND (Dental Crowns)) AND (Marginal Fit)) NOT (Systematic Review); ((CAD CAM) AND (Dental Crowns)) AND (Occlusion); ((CAD CAM) AND (Dental Crowns)) AND (Internal Fit); ((((dental crowns) AND (CAD CAM)) AND (internal fit)) NOT (implant crowns)) NOT (endocrowns); ((dental crowns) AND (CAD CAM)) AND (occlusal contact accuracy) ((dental technician) AND (dental crowns)) AND (marginal fit); (laboratory technician designed crowns) AND (accuracy); ((dental technician) AND (dental crown design)) AND (occlusal contact accuracy) ((((CAD CAM) AND (ARTIFICIAL INTELLIGENCE)) OR (AI)) AND (Dental crowns)) AND (marginal accuracy); (((((((CAD CAM) AND (Artificial Intelligence)) OR (AI)) OR (Machine Learning)) AND (Dental Technician)) OR (Lab Technician)) AND (Denta Crowns designing)) AND (Marginal Fit)
**Scopus**	(TITLE-ABS-KEY (Artificial Intelligence) OR TITLE-ABS-KEY (Machine Learning) OR TITLE-ABS-KEY (Neural network) AND TITLE-ABS-KEY (Marginal Accuracy of Dental Crowns)) AND PUBYEAR = 2025 AND (LIMIT-TO (LANGUAGE, “English”)); (TITLE-ABS-KEY (Artificial Intelligence) OR TITLE-ABS-KEY (Machine Learning) OR TITLE-ABS-KEY (Neural network) AND TITLE-ABS-KEY (Marginal Accuracy of Dental Crowns)) AND PUBYEAR = 2025 AND (LIMIT-TO (LANGUAGE, “English”)); (TITLE-ABS-KEY (Artificial Intelligence) OR TITLE-ABS-KEY (Machine Learning) AND TITLE-ABS-KEY (Marginal Accuracy of Dental Crowns)) AND PUBYEAR = 2025 AND (LIMIT-TO (LANGUAGE, “English”)); (TITLE-ABS-KEY (Artificial Intelligence) AND TITLE-ABS-KEY (Dental Crowns) AND TITLE-ABS-KEY (occlusal contact)) AND PUBYEAR = 2025 AND (LIMIT-TO (LANGUAGE, “English”)); (TITLE-ABS-KEY (Artificial Intelligence) AND TITLE-ABS-KEY (Dental Crowns) AND TITLE-ABS-KEY (accuracy assessment)) AND PUBYEAR = 2025 AND (LIMIT-TO (LANGUAGE, “English”)); (TITLE-ABS-KEY (Artificial Intelligence) AND TITLE-ABS-KEY (Dental Crowns) AND TITLE-ABS-KEY (Marginal Fit)) AND PUBYEAR = 2025 AND (LIMIT-TO (LANGUAGE, “English”)); (TITLE-ABS-KEY (Artificial Intelligence) AND TITLE-ABS-KEY (Dental Crowns) AND TITLE-ABS-KEY (Marginal Accuracy)) AND PUBYEAR = 2025 AND (LIMIT-TO (LANGUAGE, “English”)); (TITLE-ABS-KEY(CAD CAM) AND TITLE-ABS-KEY(Dental Crowns) AND TITLE-ABS-KEY(Marginal Fit)) AND PUBYEAR > 2009 AND PUBYEAR < 2026; (TITLE-ABS-KEY (CAD CAM) AND TITLE-ABS-KEY (Dental Crowns) AND TITLE-ABS-KEY (accuracy assessment)) AND PUBYEAR > 2011 AND PUBYEAR < 2026; (TITLE-ABS-KEY(CAD CAM) AND TITLE-ABS-KEY(Dental Crowns) AND TITLE-ABS-KEY(Occlusal contact)) AND PUBYEAR > 2009 AND PUBYEAR < 2026 AND (LIMIT-TO (LANGUAGE, “English”)) (TITLE-ABS-KEY(Dental Technician) OR TITLE-ABS-KEY(laboratory technician) OR TITLE-ABS-KEY(conventional techniques) AND TITLE-ABS-KEY(Dental crown designing) AND TITLE-ABS-KEY(marginal fit)) AND PUBYEAR > 2009 AND PUBYEAR < 2026 AND (LIMIT-TO (LANGUAGE, “English”)); (TITLE-ABS-KEY(Dental Technician) OR TITLE-ABS-KEY(laboratory technician) OR TITLE-ABS-KEY(conventional techniques) AND TITLE-ABS-KEY(Dental crown designing) AND TITLE-ABS-KEY(marginal accuracy)) AND PUBYEAR > 2009 AND PUBYEAR < 2026 AND (LIMIT-TO (LANGUAGE, “English”)); (TITLE-ABS-KEY(Dental Technician) OR TITLE-ABS-KEY(laboratory technician) OR TITLE-ABS-KEY(conventional techniques) AND TITLE-ABS-KEY(Dental crown designing) AND TITLE-ABS-KEY(occlusal contact)) AND PUBYEAR > 2009 AND PUBYEAR < 2026 AND (LIMIT-TO (LANGUAGE, “English”));
**Web Of Science**	TS=(“Artificial Intelligence” OR “Machine Learning”) AND TS=(“Dental crowns”) AND TS=(“Accuracy”); TS=(“AI-assisted” OR “machine learning”)) AND TS=(“marginal fit” OR “internal fit”); TS=(“Artificial Learning” OR “ Neural Network” OR “Deep Learning”) AND TS=(“Dental Crowns”) AND TS=(“Occlusal Contact”) TS=(“CAD-CAM”) AND TS=(“Dental crowns”) AND TS=(“Marginal Accuracy”); TS=(“CAD-CAM”) AND TS=(“marginal fit” OR “internal fit”); TS=(“CAD-CAM”) AND TS=(“Dental Crowns”) AND TS=(“Occlusal Morphology”) TS=(“Dental Technician” OR “laboratory technician” OR “Conventional”) AND TS=(“Dental crowns”) AND TS=(“Accuracy”); TS=(“Dental Technician” OR “laboratory technician” OR “Conventional”) AND TS=(“marginal fit” OR “internal fit”); TS=(“Dental Technician” OR “laboratory technician” OR “Conventional”) AND TS=(“Dental Crowns”) AND TS=(“Occlusal Contact Accuracy”)
**Cochrane**	(“Dental Crown” OR “Single crown” OR “Fixed dental prosthesis”) AND (“Artificial intelligence” OR “Machine learning” OR “Deep learning” OR “Neural network” OR “Generative adversarial network” OR “AI-assisted design”) AND (“CAD-CAM” OR “Computer-aided design” OR “Digital dentistry” OR “Technician-designed”) AND (“Marginal fit” OR “Marginal adaptation” OR “Internal fit” OR “Internal adaptation” OR “Occlusal contact” OR “Occlusal accuracy”)
**Lilacs**	(Artificial Intelligence) AND (Dental Crowns) AND (Marginal Accuracy); (Artificial Intelligence) AND (Dental Crowns) AND (Marginal Fit); (Artificial Intelligence) AND (Dental Crowns) AND (Accuracy assessment); (Artificial Intelligence) AND (Dental Crowns) AND (Occlusal contact morphology); (Artificial Intelligence) AND (Dental Crowns) AND (Internal Fit); (Artificial Intelligence) AND (Dental Crowns) AND (Precision); (Artificial Intelligence) OR (Machine learning) AND (Dental Crowns) AND (accuracy); (Artificial Intelligence) OR (Machine learning) AND (Dental Crowns) AND (Marginal Fit); (Artificial Intelligence) OR (Machine learning) AND (Dental Crowns) AND (Occlusal Contact); (Artificial Intelligence) OR (Machine learning) OR (Neural Network) AND (Dental crown designing) AND (Marginal Accuracy) (CAD CAM) AND (Dental Crowns) AND (Marginal Accuracy); (CAD CAM) AND (Dental Crowns) AND (Marginal Fit); (CAD CAM) AND (Dental Crowns) AND (Internal Fit)

**Table 3:** Characteristics of included studies.

Author	Study Design	Sample Size	Comparative Groups	Outcomes Evaluated	Method of Evaluation	Relevant Findings
Nejatidanesh et al., 2016 [26]	In vitro study	40 cement-retained ISCs	CAD-CAM-E-max CAD (Cerec AC system), zirconia-based (Cercon system) v/s conventional technique—IPS e-max Press, and metal–ceramic restorations.	Accuracy, marginal/internal fit	Silicone replica technique and stereomicroscope	All restorations showed clinically acceptable gaps, with CAD/CAM crowns exhibiting better marginal fit.
Mostafa et al., 2018 [27]	In vitro study	45 lithium disilicate crowns	Digital imaging and digital manufacturing (DD), digital imaging and pressing (DP), and traditional impression and pressing (TP).	Marginal fit in terms of marginal gap (MG) and vertical gap (VG)	Microcomputed tomography (micro-CT)	Compared with the DP and TP groups, the DD group exhibited significantly lower vertical MG; nevertheless, mean values across all groups were clinically acceptable.
Cheng et al., 2021 [28]	Randomized clinical trial (RCT)	40 provisional crowns	Interim single crowns (SCs) fabricated via conventional procedure and CAD-CAM.	Prosthesis fabrication time, marginal fit, proximal contact, occlusal contact and crown morphology	Clinical evaluation	The digital workflow produced better occlusal contacts, with no significant differences in marginal fit, proximal contact, or crown morphology compared with the conventional workflow.
Cho et al., 2023 [29]	In vitro study	30 datasets	AI-based deep learning generative adversarial network (GAN)-based software (Dentbird Crown; Imagoworks Inc.) vs CAD-CAM (3Shape Dental System; 3Shape)	Time efficiency, occlusal morphology, internal fit	Superimposition analysis	Compared with conventional software, the GAN-based AI method demonstrated greater time efficiency, reduced occlusal morphology deviation after optimization, and improved internal fit.
Ding et al., 2023 [30]	In vitro study	600 datasets	AI-based3D deep convolutional generative adversarial network (3D-DCGAN), natural tooth (NT), CEREC bio generic individual design (BI), and technician CAD (TD)	Cusp angle, 3D similarity, occlusal contact, and dynamic finite element assessment (FEA)	Superimposition via Geomagic Software	3D-DCGAN-generated crowns showed greater similarity to NT morphology and biomechanics than BI and TD designs.
Bae et al., 2023 [31]	In vitro study	360 zirconia crowns	3 CAD software—EZIS VR (DDS, Seoul, Korea), 3Shape Dental System (3Shape, Copenhagen, Denmark), and Exocad (Exocad, Darmstadt, Germany).	Fit and trueness	Silicone replica technique and 3D metrology software	Fit and trueness varied with the CAD/CAM system used; all systems produced clinically acceptable results.
Cho et al., 2024 (J Dent 141) [32]	In vitro study	30 datasets	AI-assisted deep learning (DL)-based two software—anatomy aware (AA) and automated design (AD) v/s CAD-CAM based designing (NC).	Tooth morphology, internal fit, occlusion, proximal contact	3D geometric analysis	DL-based dental software produced crowns with optimized morphology, internal fit, cusp angle, and occlusal contacts, requiring minimal modification and representing a viable alternative to technician-based posterior crown design.
Cho et al., 2024 (J Dent 147) [33]	In vitro comparative digital study	20 resin-based casts for implant-supported crowns (ISCs)	Conventional CAD/CAM vs AI-based utilizing DL software.	Morphology, occlusal contact, emergence profile, proximal contacts	Modeling software (PowerShape; Autodesk) and inspection software (Geomagic Control X; 3D Systems)	A DL-based method enables efficient posterior ISC design with morphological outcomes comparable to conventional CAD-CAM.
Kızılkaya et al., 2024 [34]	In vitro study	30 provisional crowns	CAD software programs: Dentbird, Exocad and Inlab 20.	Marginal fit and internal fit	3D analysis software	Dentbird CAD software program provided the most accurate fit values that closely matched the design.
Nagata et al., 2025 [16]	In vitro study	20 crowns	AI-assisted CAD v/s conventional CAD.	Design time, marginal fit, proximal contact intensity	Superimposition via Geomagic Software	AI-assisted CAD significantly reduced design time and maintained accurate fit and occlusal outcomes vs non-AI CAD.
Ren et al., 2025 [35]	Digital simulation study	291 casts obtained from patients requiring ISC	CAD-CAM (ExoCad and 3Shape) based v/s AI-generated v/s technician designed-crowns	Fit, morphology, occlusal, and proximal contact accuracy	3D surface superimposition, digital gap analysis, and virtual contact evaluation techniques	AI-generated implant-supported crowns more closely matched clinically validated technician designs than conventional CAD crowns, particularly in contour, occlusal morphology, and emergence profile.
Win et al., 2025 [36]	Prospective comparative clinical study	124 provisional crowns	3D generative artificial intelligence design (GAID) v/s conventional computer-aided design (CCAD) method.	Fit accuracy	Triple-scan technique	AI-designed crowns demonstrated comparable fit accuracy relative to conventional crowns.

**Table 4:** Summary of marginal and/or internal fit/adaptation and occlusal contact accuracy of single crowns designed via AI, CAD-CAM, and technician (conventional procedure).

Author (Year)	Comparison Group	Marginal Fit/Accuracy and/or Internal Fit (µm)	Occlusal Contact Accuracy (points/mm^2^/RMS)	Overall Conclusion
Cho et al., 2023 [29]	AI v/s CAD-CAM	AI-designed crowns have better internal fit (55.4 ± 17 µm) than CAD-CAM designed (85.6 ± 29.6 µm).	Not recorded.	AI-assisted crown design demonstrates a statistically superior internal fit to the prepared abutment compared with CAD-CAM-based design workflows.
Ding et al., 2023 [30]	AI v/s CAD-CAM	Not recorded.	The number and area of contact points measured with 100 μm and 200 μm articulating papers were similar for AI- and CAD-CAM-generated crowns, showing no significant differences.	Occlusal contact points and areas for 3D-DCGAN and CAD-CAM crowns are comparable and closely replicate the occlusal relationships of natural teeth.
Cho et al., 2024 (J Dent 141) [32]	AI v/s CAD-CAM	The AA group showed the largest internal gap (lowest fit, *p* < 0.001), while the AD group had the smallest gap (highest fit), not significantly different from the NC group (*p* = 0.037).	AI-based crowns showed 44 (10.3%) & 37 (8.6%) of the planned contacts (+20 μm to 0 μm;), whereas CAD-CAM crowns showed 83 (19.4%). Heavy premature contact (less than −20 μm,) was 22 (5.1%) & 2 (0.5%) for AI-based crowns and 5 (1.2%) for CAD-CAM crowns.	DL-based software-generated crowns with internal fit and occlusal contact accuracy comparable to those of conventional CAD-CAM designs, requiring minimal clinical adjustment.
Cho et al., 2024 (J Dent 147) [33]	AI v/s CAD-CAM	Not recorded.	DL-based crowns showed 31.5% (75) of planned contacts (+20 μm to 0 μm;), whereas CAD-CAM crowns showed only 21.6% (62) of planned contacts. Light contacts (0 μm to −20 μm) were more with CAD-CAM crowns (13.4%—32 contacts) as compared to DL-designed crowns (1.3%—3 contacts).	DL-based crowns achieve more precise occlusal contacts than CAD-CAM crowns, reducing occlusal discrepancies and the need for adjustments.
Kızılkaya et al., 2024 [34]	AI v/s CAD-CAM	Palatal and distal marginal fit differed significantly between AI- and CAD-CAM-based crowns (*p* < 0.05), whereas buccal and mesial surfaces showed no significant differences. Internal fit differed significantly for occlusal, buccal, and distal surfaces (*p* < 0.05), but not for palatal or mesial surfaces (*p* > 0.05).	Not recorded.	The best fit values were observed for the AI-based Dentbird software program.
Nagata et al., 2025 [16]	AI v/s CAD-CAM	No significant difference was observed for marginal fits between AI-based and CAD-CAM-based crowns.	The occlusal surface accuracy measured 275.5 ± 116.8 μm for the conventional CAD system, compared with 25.7 ± 13 μm for the AI-enabled CAD system.	AI-designed crowns demonstrated favorable occlusal surface accuracy and satisfactory marginal fit.
Win et al., 2025 [36]	AI v/s CAD-CAM	Equivalence analysis demonstrated negligible marginal fit differences between AI and CAD-CAM-based crowns across regions, with mean differences of −2.8 μm (buccal), −1.16 μm (mesial), and 2.0 μm (distal). For internal fit, the mean internal gap across all surfaces ranged from 82 to 98 μm for both AI-based and CAD-CAM-designed crowns.	AI-designed crowns showed greater occlusal contact discrepancies (149 ± 66 μm) than CAD-CAM fabricated crowns (105 ± 63 μm).	Generative AI-designed crowns showed clinically acceptable fit accuracy comparable to conventional CAD designs; however, occlusal contact discrepancies were greater in the AI-designed crowns.
Ren et al., 2025 [35]	AI v/s CAD-CAM v/s technician-designed	Not recorded.	Most AI-designed crowns (16/20) showed clinically acceptable adjustable contacts with few premature contacts (3/20), comparable to technician-designed crowns, whereas CAD-CAM crowns exhibited substantially more premature contacts (3Shape: 10/20; Exocad: 17/20).	AI-assisted crown design provides occlusal contact accuracy comparable to technician-generated designs and significantly superior to CAD-CAM-generated crowns.

µm = micrometers, used for linear measurements of marginal and internal gaps, RMS = Root Mean Square deviation, representing surface deviation from the reference model in micrometers, FEA = finite element analysis, used to assess mechanical performance.

**Table 5:** Risk-of-bias assessment using Modified CONSORT checklist for assessing in vitro studies.

Author	1	2	3	4	5	6	7	8	9	10	11	12	13	14	Overall Risk of Bias
Nejatidanesh et al., 2016 [26]	Yes	Yes	Yes	Yes	No	No	No	No	No	Yes	Yes	Yes	Yes	No	Moderate–High
Mostafa et al., 2018 [27]	Yes	Yes	Yes	Yes	No	No	No	No	No	Yes	Yes	Yes	Yes	No	Moderate–High
Bae et al., 2023 [31]	Yes	Yes	Yes	Yes	No	Unclear	No	No	No	Yes	Yes	Yes	Yes	No	Moderate
Cho et al., 2023 [29]	Yes	Yes	Yes	Yes	No	No	No	No	No	Yes	Yes	Yes	Yes	No	Moderate
Ding et al., 2023 [30]	Yes	Yes	Yes	Yes	No	No	No	No	No	Yes	Yes	Yes	Yes	No	Moderate
Cho et al., 2024 (J Dent 141) [32]	Yes	Yes	Yes	Yes	No	Unclear	No	No	No	Yes	Yes	Yes	Yes	No	Moderate
Cho et al., 2024 (Implant; J Dent 147) [33]	Yes	Yes	Yes	Yes	No	Unclear	No	No	No	Yes	Yes	Yes	Yes	No	Moderate
Kızılkaya & Kara, 2024 [34]	Yes	Yes	Yes	Yes	No	No	No	No	No	Yes	Yes	Yes	Yes	No	Moderate
Nagata et al., 2025 [16]	Yes	Yes	Yes	Yes	No	Unclear	No	No	No	Yes	Yes	Yes	Yes	No	Moderate
Ren et al., 2025 [35]	Yes	Yes	Yes	Yes	No	Unclear	No	No	No	Yes	Yes	Yes	Yes	No	Moderate

1—Objective, 2—Background, 3—Intervention, 4—Outcomes, 5—Sample Size, 6—Randomization, 7—Allocation Concealment, 8—Implementation, 9—Blinding, 10—Statistics, 11—Outcomes Reported, 12—Limitations, 13—Funding, 14—Protocol.

**Table 6:** Risk-of-bias assessment done for Cheng et al. [28] using JBI tool.

S.No.	JBI Tool	Assessment (Yes/No/Unclear)
1	Was true randomization used for assignment of participants to treatment groups?	Yes
2	Was allocation to treatment groups concealed?	Unclear
3	Were treatment groups similar at the baseline?	Yes
4	Were participants blind to treatment assignment?	No
5	Were those delivering treatment blind to treatment assignment?	No
6	Were outcomes assessors blind to treatment assignment?	Unclear
7	Were treatment groups treated identically other than the intervention of interest?	Yes
8	Was follow up complete and if not, were differences between groups in terms of their follow up adequately described and analyzed?	Yes
9	Were participants analyzed in the groups to which they were randomized?	Yes
10	Were outcomes measured in the same way for treatment groups?	Yes
11	Were outcomes measured in a reliable way?	Yes
12	Was appropriate statistical analysis used?	Yes
13	Was the trial design appropriate, and any deviations from the standard RCT design (individual randomization, parallel groups) accounted for in the conduct and analysis of the trial?	Yes
	Overall Assessment	Moderate Risk

**Table 7:** Risk-of-bias assessment done for Win et al. [36] using JBI tool.

S.No.	JBI Item	Assessment (Yes/No/Unclear)
**1**	Is it clear in the study what is the “cause” and what is the “effect”?	Yes
**2**	Were the participants included in any comparisons similar?	Yes
**3**	Were the participants included in any comparisons receiving similar treatment/care, other than the exposure or intervention of interest?	Yes
**4**	Was there a control group?	Yes
**5**	Were there multiple measurements of the outcome both pre and post the intervention/exposure?	No
**6**	Was follow-up complete and if not, were differences between groups in terms of their follow-up adequately described and analyzed?	Unclear
**7**	Were the outcomes of participants included in any comparisons measured in the same way?	Yes
**8**	Were outcomes measured in a reliable way?	Yes
	**Overall Assessment**	**Low Risk**

## Data Availability

The data that support the findings of this study are available from the corresponding author upon reasonable request.

## Supplementary Materials

The following supporting information can be downloaded at: https://www.mdpi.com/article/10.3390/medicina62030567/s1, Table S1: PRISMA 2020 checklist [59].
